# From plant immunity to food security: an interview with Ksenia Krasileva

**DOI:** 10.1186/s12915-018-0597-1

**Published:** 2018-11-01

**Authors:** Ksenia Krasileva

**Affiliations:** Department of Plant and Microbial Biology, University of California, Berkley, CA 94720 USA

**Keywords:** Plant pathogen, Food security, NLRs, Plant immunity, Plant genomics

## Abstract

Ksenia Krasileva is an Assistant Professor at UC Berkley, studying innate immunity in plants. Ksenia’s work combines plant genomics and plant-microbe interactions with new technologies, spanning basic studies and translational research in agriculture. In this interview Ksenia shares her experience with research and leading a lab, as well as thoughts on innovations in publishing.

## What are the questions driving your research?

The major question that drives our research is how plants, having only an innate immune system, are able to recognize new pathogens and withstand diseases. I believe that understanding the information encoded in plant genomes and how genomic diversity is generated will help to answer this question. We are particularly interested in plant immune receptors called NLRs, which are very flexible protein platforms. We hope that in the future we can start engineering NLRs and thus protect crops from epidemics.

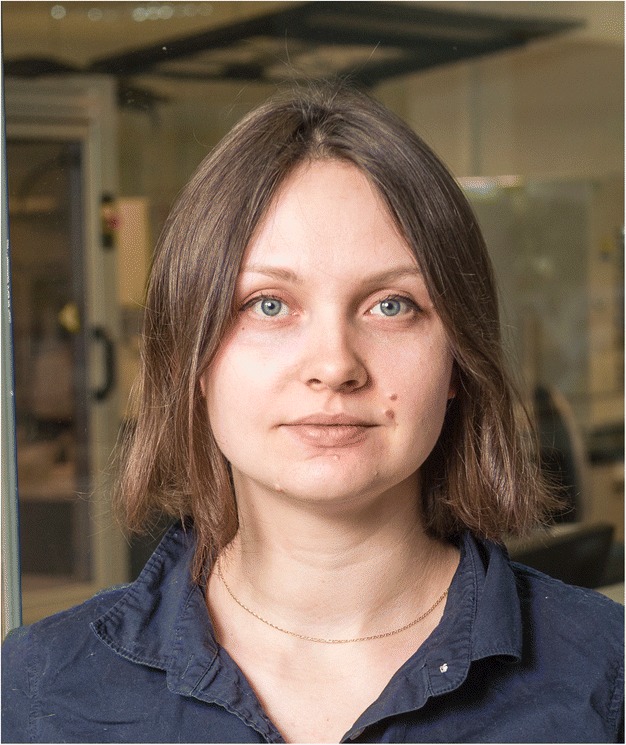



## Looking back, is there a project that your lab pursued that stands out for you as particularly inspiring, tough, or simply memorable?

One very memorable project is actually the one published in *BMC Biology* in 2016, a study where we first compared NLRs across 50 plant genomes and uncovered NLRs that fuse with other plant proteins [1]. We did our first comparative genomics survey identifying NLR fusions in 2013 and, for a few years, people did not see the significance of this finding and attributed such novel fusions to mis-annotations. These data were still unpublished when major breakthroughs occurred in the functional characterization of such fusions: in two RRS1 from *Arabidopsis thaliana* and RGA5 and Pi-k from *Oryza sativa* (rice), leading to the integrated ‘decoy’ model of pathogen recognition [2]. Now we know that such proteins, which we named “NLRs with integrated domains” or NLR-IDs, can act as ‘baits’ for the pathogens. The breadth of information we obtained with this comparative genomics screen continues to fuel grants and projects in my lab.

## Is there a paper or a scientist that inspired you, or was seminal for your research?

There are many scientists who inspired and continue to inspire me, among them Michael Eisen, Lior Pachtor, Kimmen Sjolander, Eugene Koonin, Doreen Ware, Magnus Nordborg, Anna-Liisa Laine… the list can continue for several pages, and of course people who had direct impact were my mentors: Steven Lindow, Brian Staskawicz, and Jorge Dubcovsky. One scientist who has had the most profound impact on my research and my development as a scientist has been Sophien Kamoun. I first met Sophien as a second year graduate student, and his energy and enthusiasm about research were contagious. Reading Sophien’s early reviews such as on the host resistance to *Phytophthora* [3] showed me how to keep scientific writing concise and clear.

## What are your guiding principles for running a lab? Do you have any advice to share with our readers?

My guiding principles are honesty, positive attitude, and also grit and following research through. Many aspects of research, especially when it is new, can be overwhelming, so my advice is to put thoughts down on paper, whether it is an idea, new technique, or a ‘to do’ list. It helps to clear the head but also to formulate exactly what needs to be done and form a plan how to do it.

## If you could, what would you tell your younger self?

To spend less time worrying. That everyone makes a lot of mistakes, and this is part of learning and becoming good at what you do. Also, in research the most important thing is your research team, and it pays to invest time in putting it together.

## What are the most urgent questions you’d like to see addressed in your field?

We need to come up with rapid responses to plant pathogens that will allow us to tackle the full circle of sequencing a pathogen from the field, identifying molecules that we can target, and the developing resistance genes, all in a timeframe comparable to how animal vaccines, such as the flu vaccines, are developed now. Once a pathogen is detected, we should aim for resistant plants to be ready to go in the field in 1–2 years.

## Are there any innovations in publishing you’d like to see happen?

I am a strong supporter of bioRxiv and open access, and I am happy to see how much has happened in publishing already over the past few years. It was fantastic to see the pledge from journals signed last week to publish reviews alongside papers. We are moving in the right direction. What would be great to see is a full history of a paper, from bioRxiv to journal submissions to publication, where was it submitted first, was it accepted/rejected, and what were the reviews, what were the improvements, and where did it go next. And of course, in an ideal world, scientists should not be worried about the cost of publication, but this could be hard to achieve for practical reasons. Personally, I liked the model from the Biotechnology and Biological Sciences Research Council (BBSRC), which is a major funding agency in the UK. BBSRC set up separate funds to cover publication costs in my previous institute and included additional publication funds in PhD fellowships; this meant that I did not have to pay publishing fees at the expense of research funds and could keep all papers as open access.

### Website:

https://krasilevalab.org

### Twitter:

@kseniakrasileva
